# Use of Fluorescence In Situ Hybridization (FISH) in Diagnosis and Tailored Therapies in Solid Tumors

**DOI:** 10.3390/molecules25081864

**Published:** 2020-04-17

**Authors:** Natalia Magdalena Chrzanowska, Janusz Kowalewski, Marzena Anna Lewandowska

**Affiliations:** 1 Molecular Oncology and Genetics Department, Innovative Medical Forum, The F. Lukaszczyk Oncology Center, 85-796 Bydgoszcz, Poland; chrzanowskan@co.bydgoszcz.pl; 2 Department of Thoracic Surgery and Tumors, Ludwik Rydygier Collegium Medicum in Bydgoszcz, Nicolaus Copernicus University, 85-067 Torun, Poland; kowalewskij321@cm.umk.pl

**Keywords:** personalized medicine, targeted treatment, FISH, *HER2*, *ALK*, *ROS1*, personalized oncology, t(X,18), *COL1A1-PDGFB*

## Abstract

Fluorescence in situ hybridization (FISH) is a standard technique used in routine diagnostics of genetic aberrations. Thanks to simple FISH procedure is possible to recognize tumor-specific abnormality. Its applications are limited to designed probe type. Gene rearrangements e.g., *ALK*, *ROS1* reflecting numerous translocational partners, deletions of critical regions e.g., 1p and 19q, gene fusions e.g., *COL1A1-PDGFB*, genomic imbalances e.g., 6p, 6q, 11q and amplifications e.g., *HER2* are targets in personalized oncology. Confirmation of genetic marker is frequently a direct indication to start specific, targeted treatment. In other cases, detected aberration helps pathologists to better distinguish soft tissue sarcomas, or to state a final diagnosis. Our main goal is to show that applying FISH to formalin-fixed paraffin-embedded tissue sample (FFPE) enables assessing genomic status in the population of cells deriving from a primary tumor or metastasis. Although many more sophisticated techniques are available, like Real-Time PCR or new generation sequencing, FISH remains a commonly used method in many genetic laboratories.

## 1. Introduction of In Situ hybridization

Screening by in situ hybridization plays a supportive role in personalized medicine. A wide range of recognized aberrations: rearrangements resulting from translocations, insertions or inversions, losses (deletions) and gains (e.g., amplification) can be evaluated mainly using fluorescence in situ hybridization (FISH). In certain cases, chromogenic in situ hybridization (CISH) is introduced into the molecular pathology and molecular oncology field. Both in situ hybridization methods are based on the same idea of annealing to the region of interest, detection, assessment and spatial localization [1]. The main difference between the methods is the method of genomic region labeling, either with biotin or digoxigenin (CISH) or with fluorescent tags (FISH), followed by an appropriate detection system. For probes labeled with biotin, streptavidin conjugated with horseradish peroxidase (HRP-streptavidin) is used for detection. For probes with attached digoxigenin, an anti-digoxigenin fluorescein primary antibody, followed by an HRP-conjugated anti-fluorescein secondary antibody are used, and signals are counted under a bright-field microscope [2,3]. FISH detection is direct and requires fluorescence microscopy. Testing of genetic markers often plays a pivotal role in making decisions concerning patients, from patient risk stratification to implementing the appropriate treatment. Both in situ techniques are used in this field. For example, compared with FISH, CISH has been shown to have a sensitivity of 97.5% and a specificity of 94% for detection of the *HER-2*/neu gene amplification [4]. The concordance rate between FISH and CISH results calculated in a group of 4460 patients diagnosed with breast cancer was at the level of 96%, showing CISH to be a comparable technique to FISH. Most sources are in agreement and report almost equal performance of FISH and CISH in gene amplification assays. However, CISH may show lower sensitivity for low-level amplifications due to the presence of polysomy of chromosome 17 [2]. Similar observations were presented for *ALK* (*ALK* receptor tyrosine kinase) rearrangements tested using FISH and CISH. Testing 449 samples indicated 19 and 18 *ALK*-positive samples, respectively, in a comparison of these two methods. CISH sensitivity was at a level of 94.4% and specificity at 100%. Although every diagnostic tool, not only based on hybridization, but also on protein expression, such as immunohistochemistry (IHC), has advantages and disadvantages, the FISH technique is currently stated as the reference method [5]. A detailed comparison of the three methods is presented in Table 1.

An alternative approach to in situ testing is the use of mRNA as a biomarker. The technique uses a single-stranded DNA probe complementary to the tested mRNA molecule. Standard protocol includes digestion, hybridization, detection using an anti-digoxigenin antibody and an HRP-labeled secondary antibody, and result visualization using a chromogen, e.g., DAB (3,3′-diaminobenzidine) and a bright-field microscope. mRNA ISH is faster, cheaper, easier in preparation and less toxic in comparison with the above mentioned ISH techniques. A ready-to-use kit can be used for the *HER2* gene expression assessment in breast cancer on formalin-fixed paraffin-embedded (FFPE) samples. The main limitation, as in other techniques based on mRNA assessment, is the poor stability of ribonucleic acid [3,6].

## 2. Fluorescence In Situ Hybridization In Solid Tumors

Fluorescence in situ hybridization is a cytogenetic-molecular technique developed in the 1980s. The standard protocol of FISH carried out on formalin-fixed paraffin-embedded (FFPE) tissue begins with a selection of the representative population of tumor cells by a pathologist who marks a section for FISH analysis on a Hematoxylin and Eosin (H&E)-stained histopathological tissue sample. A crucial issue at this pre-analytical step is the percentage of tumor cells in the sample, since a low percentage may lead to an uninformative result of FISH scoring and the need to repeat the whole procedure, starting from the selection of a new FFPE section. In the following step, an unstained sliced histological sample undergoes a standard procedure of deparaffinization and rehydration, consisting of heating the slide in a cabinet pre-warmed to 60 °C and immersing the slide in a series of wells with xylene and absolute ethanol. Subsequently, incubation with a pretreatment solution is followed by digestion using a protease solution. Incubation time is optimized individually for every FISH probe protocol. This procedure enables removing chemicals used previously to provide the best conditions for maintaining cell integrity as well as DNA structure. The nucleic acid bereft of cross-links can easily bind with a complementary sequence of the probe, significantly improving the efficiency of hybridization. Some protocols require the use of hydrochloric acid (HCl) and additional washing in saline-sodium citrate (SSC). The FISH protocol includes the following steps: denaturation of cellular DNA of the sample and the probe into single strands and hybridization of the probe with a target nucleic sequence. Fast-working hybridization buffers shorten this step significantly from an overnight incubation to a few hours. The final steps of the procedure are post-hybridization washes in SSC solutions of enriched with non-ionic detergent (NP-40) which reduce unspecific signals of the unbound probe. The final analysis of the FISH slide involves detection using an epifluorescence microscope equipped with an adjusted set of filters [8,34,35,36,37].

New approaches to FISH preparation include automated systems in which the whole procedure may be performed by a device, e.g., Ventana Medical System (Tucson, AZ, USA), with a slight support from a laboratory technician. This approach spares time and eliminates exposure to harmful chemicals, such as xylene which is used in the manual procedure.

FISH results are obtained by counting hybridization signals of the probe in each cell. Every laboratory should define its own counting procedure including the number of analyzed cells, the percentage of re-scoring of cells by a second diagnostician, control slides, cut-off for an abnormal result. Although counting signals is mostly still performed in a manual way, there are automatic counting systems available as well. Such software uses algorithms programmed to search for objects with the required shape (cells) and the presence of fluorescence signals, which are recognized as bright dots and then counted. This approach is based on an analysis of photographs, taken by a diagnostician, of representative fields with neoplastic cells. Every field is verified, which includes exclusion of non-cell objects, correction of the inaccurately marked shape of cells and verification of recognized signals. The final result is presented as the abnormal cell percentage (as for break-apart probes, e.g., for genes *ALK*, *ROS1*, *EWSR1*) or ratio (for HER2/CEP17, 1p/1q, 19q/19p) [35,38].

Modern concept of FISH technique present microfluidic platforms which are dedicated to the analysis of circulating tumor cells (CTCs). The cells can be obtained from the patient blood sample and used as a tumor biomarker in diagnosing and metastasis prognosis. Cellular DNA applied on a microarray is put into microchannel what enables flow-based incubation with chemicals. In this manner kinetics of hybridization process between probe and target sequence is improved. This significantly enhances signal strength. Moreover, the method improved reproducibility and reduce labor time. The method has been tested for genes *HER2*, *KMT2A* (lysine methyltransferase 2A) and others. The way of receiving CTCs seems to be the biggest challenge of the microfluidic platform [35,39].

## 3. Types of Probes Available in FISH Technique

**Figure 1:**
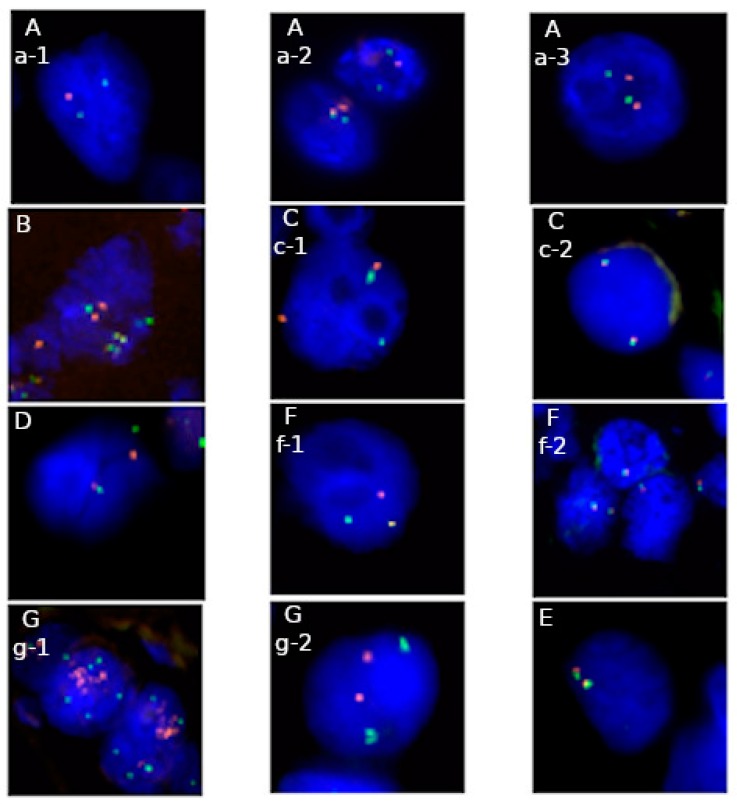
Applications of fluorescence in situ hybridization (FISH) in genetic diagnostics in solid tumors on FFPE material: **A**—1p/19q probe: a-1 deletion of 1p32 *locus*, a-2 normal signal pattern (cell on the left) and deletion of 19q13 *locus* (cell on the right), a-3 normal signal pattern (Abbott Molecular), **B**—dual fusion probe: fusions and normal signals pattern of *COL1A1* and *PDGFB loci* (ZytoVision)*,* **C**—break apart probe: c-1 rearrangement of *ALK* gene, c-2 normal signal pattern (Abbott Molecular), **D**—break apart probe: rearrangement of *EWSR1 locus* (Abbott Molecular), **F**—break apart probe: f-1 rearrangement of *ROS1* gene, f-2 normal signal pattern (Empire Genomics), **G**—*locus* specific probe: g-1 amplification of *HER2 locus*, g-2 normal signal pattern (Abbott Molecular), **E**—break apart probe: normal signal pattern of *SS18 locus* (Abbott Molecular). Majority probes indicate region of interest in red color and control region—in green, excluding picture B, where red color indicates *COL1A1* gene *locu*s, green color—*PDGFB* gene *locus*, yellow color—fusion of *COL1A1-PDGFB* and *PDGFB-COL1A1.* Full names of available commercial probes are presented in Table 2.

A significant issue in the FISH technique is the choice of the appropriate probe which determines the value of a test for a patient (Table 2). Every type of FISH probe enables detecting different chromosomal aberrations. Examples of practical use of a different type of FISH probes present Figure 1A–G. *Locu*s-specific probes may be used to assess the number of extra copies of the target. Establishing the status of amplification requires two differently labeled *loci*, target and control regions. The decisive factor for amplification is the ratio parameter. It is a quotient of the total number of red-labeled tested gene signals divided by the total number of green-labeled control sequence signals. A minimum result of 2.0 confirms amplification [12,36].

*Locus*-specific probe can also be used to assess the loss of a chromosome sequence in a genome. As previously said, a probe consists of two differently marked regions, target and control *locus*. Deletion is reported when the number of cells with dominating control *locus* signals exceeds that of cells with dominating target *locus* signals. The cut-off should be calculated for each histological sample separately [12].

Different from the above-mentioned types of probes are those used for detecting chromosomal rearrangements. One of these probes, the dual-color probe, encompasses two gene targets. Due to the breaks within these targets and exchange between their halves, two fusions arise, which are the proof of translocation at the chromosome level. In the case of the split type probe, two contrast fluorescent dyes are complementary to two distant ends of the same gene, staying in close relation to the most frequent breakpoint the target [12].

## 4. Validation of FISH Method

Standards and guidelines for chromosome studies of lymph node- and solid tumor-acquired chromosomal abnormalities using FISH assays, developed for clinical laboratory geneticists on behalf of the American College of Medical Genetics and Genomics (ACMG) Laboratory Quality Assurance Committee, have not changed since their last revision in 2018 [43]. The result of the first counting assessment should be verified by a second diagnostician to assure reproducibility, increase the analytical reliability of FISH results and reduce the risk of errors [38]. Furthermore, automation of the in situ hybridization process and advanced software may significantly improve the quality of work. It has been estimated that the upper cut-off for normal results in a FISH test should be determined by calculating the 95% confidence level for the probe signal patterns detected in representative normal control samples [34]. As a good practice, laboratories should regularly participate in external quality assessment [44].

## 5. FISH Analysis in Solid Tumors

### 5.1. Lung Cancer

NSCLC (non-small cell lung cancer) is an example of a tumor with well-known genetic and epigenetic mechanisms of carcinogenesis. The Cancer Genome Atlas project has helped to identify the most common genomic alterations in NSCLC which include point mutations and structural rearrangements in protooncogenes, such as: *EGFR* (epidermal growth factor receptor), *KRAS* (KRAS proto-oncogene, GTPase), *BRAF* (B-Raf proto-oncogene serine/threonine kinase), *MET* (MET proto-oncogene, receptor tyrosine kinase), *ALK, RET* (ret proto-oncogene), *ROS1* (ROS protooncogene 1, receptor tyrosine kinase), *MYC* (MYC proto-oncogene, bHLH transcription factor). For example, mutations in the *EGFR* gene occur in up to 15% of European patients [45] and in 9%–10,62% of Polish patients [46,47,48]. Moreover, rearrangements, within the most common *ALK* kinase, in general, account for around 3%–7% of cases if the most frequent partner, the *EML4* (EMAP like 4) gene, is involved. This situation accounts for 2.7%–7.5% [49,50,51] and 2.82%–4.5% [40,52] of European and Polish patients, respectively.

### 5.2. ALK Gene

There are three ways of *ALK* activation. The first one, believed to be the original event in the NSCLC oncogenesis, involves numerous structural rearrangements of the short arm of chromosome 2, including gene *locus* 2p23. The inversion inv (2) (p21p23) results in gene fusion between the *ALK* and *EML4* genes. For the *EML4-ALK* fusion, at least 15 different variants have been recognized. What is more, this chimeric transcript is only one of 22 possible fusion products [18,53]. Other potential translocational partners include the following genes: *KLC1* (kinesin light chain 1) [54], *KIF5B* (kinesin family member 5B) [55], *TFG* (trafficking from ER to Golgi regulator) [56]. The breakpoint of the rearranged *ALK* gene lies in the kinase domain, frequently within exon 20 [53].

The second activating mechanism are gains of the *ALK locus*. Visually, in the FISH analysis, they are observed as extra copies of un-rearranged *ALK loci*. They differ from polysomy by the presence of at least 10 gene copies per cell in at least 10% of the analyzed cell population [16,40]. They are observed in a significant percentage of cells, ranging from 50% to 95%, in up to 63% of patient samples [16].

The third known activating mechanism are mutations. Mutations in the *ALK* gene, such as L1196M, modify the protein structure which prevents the protein from binding drug molecules. *ALK* mutations are responsible for TKI (tyrosine kinase inhibitors) treatment resistance in about 25%–30% of cases, while *ALK* amplification in 15% [18,53]. The last two mechanisms are observed in patients with secondary resistance to ALK-TKI, such as crizotinib. The co-existence of both of them is possible [18].

Detection of an *ALK* rearrangement or gain is possible with the FISH technique. The method is sufficient to detect numerous fusion variants thanks to the special structure of the probe—two differentially labeled short DNA sequences that flank breakpoints in the *ALK* gene. The short distance between these red and green parts of the probe yields a picture of overlapped signals or a fusion signal. The rearrangement is recognized when the distance between the 5′ and 3′ parts exceeds two diameters of the signal. This principle may sometimes cause uncertainty in the analysis, therefore passing the external quality control assessment is vital. The result is stated as truly positive when more than 25 cells out of 50 (50%) counted from at least four microscopic fields indicate the above-described separation of red and green signals in at least one gene fusion. When the abnormality is observed in 5–25 cells (10%–50%), the result is considered equivocal. Analysis done by a second analyst on 50 new cells qualifies the result as positive when the abnormality occurs in more than 15 cells (15%) out of the total 100 analyzed [40].

Identification of genetic abnormalities of the *ALK* gene (conducted in association with established therapies) is confirmed by the U.S. Food and Drugs Administration (FDA)-validated and approved tests, including FISH. The potential of *ALK* testing extends to searching for possible mechanisms of crizotinib resistance, which is believed to be related to not only mutations, but also fusion amplification, for example, *EML4-ALK*. Therefore, the diagnostics of this marker may be helpful in the development of next-generation *ALK* inhibitors [57].

### 5.3. ROS1 Gene

Another tumorigenesis pathway in NSCLC are rearrangements in the *ROS1* gene. Disruptions of this gene have been identified for the first time in glioblastoma cell lines [57,58,59]. *ROS1* rearrangements can play a role in targeted treatment as important as *ALK*-ones, although they represent only 0.9% adenocarcinomas and 2% of NSCLC cases [7,60]. The *ROS1* gene is involved in translocations with other genes, located on many chromosomal *loci*. Currently, 14 different gene partners have been determined, for instance: *EZR* (ezrin) [61,62,63], *GOPC* (Golgi associated PDZ and coiled-coil motif-containing) [64], *KDELR2* (KDEL endoplasmic reticulum protein retention receptor 2) [65], *LRIG3* (leucine-rich repeats and immunoglobulin-like domains 3) [59], *SDC4* (syndecan 4) [62,66], *TPM3* (tropomyosin 3) [62], and others. In every such translocation, including rearrangement with the *SLC34A2* (solute carrier family 34 member 2) gene and the most frequent one with the *CD74* gene [56], the telomeric part of the *ROS1* gene in transferred to the fusion partner gene or is deleted. Consequently, activated kinase enhances proliferation of tumor cells, while also protecting them from death [7,67].

Assessing the *ROS1* gene status is possible thanks to FISH using a break-apart probe. Similarly to the *ALK* gene, a positive result is established if the separation of both differently labeled ends of the probe is greater than two diameters of a signal, or a deletion of the 5′ end of the gene is observed next to at least one non-rearranged signal. *ROS1* gene rearrangement has to occur in at least 15% of the analyzed population of nuclei to assure that patient is *ROS1* positive [7].

According to the newest recommendations released by the NCCN (National Comprehensive Cancer Network) issued in 2018, testing of *ROS1* rearrangements is required prior to the use of TKI inhibitors, as it is in *ALK*-positive patients. Crizotinib used in metastatic and advanced NSCLC significantly improves response rate [68,69].

### 5.4. c-MET Gene

In lung cancer, as well as in other tumors such as kidney, stomach, colon and NSCLC, overexpression of the *c-MET* gene is observed. The *c-MET* protooncogene belongs to transmembrane proteins which, after binding a ligand–HGF (hepatocyte growth factor) for *c-MET*, activate the *MAPK* (mitogen-activated protein kinases) signaling pathway [70]. There are a few possible ways of activation of the gene, for instance: amplification, activating mutations, transcriptional regulations and auto- or paracrine regulation related with the ligand [71]. When extra copies of the gene *locus* and/or mutation of the gene arise, this signal becomes enhanced which leads to a stronger cell proliferation and blockade of apoptosis, and consequently promotes neoplastic progression. Each of these alterations results in a permanent phosphorylation of the protein and, consequently, its constitutive activation [72].

Gains of the *c-MET locus* (≥5 copies/nucleus) occur in NSCLC with a frequency of 8%–10% [73,74]. Recognition of abnormality type as copy number aberration versus amplification depends on the technique used. In MLPA (multiplex ligation probe amplification), gain of the c-*MET locus* occurs in 7% NSCLC, while amplification, regarded as ≥4 copies, in 5% of cases [75]. Assessing the number of *c-MET* gene copies in FISH is possible thanks to probes complementary to the tested gene *locus* and control region of the centromere of chromosome 7 [76]. Microscopic analysis of about 100 tumor cells confirms amplification if the ratio result for *c-MET*/centromere 7 is ≥2 or if more than four copies of the *c-MET locus* are observed [71,72]. According to other references, 80% of cells should present more than 6 copies of the gene and a ratio result exceeding 2 [76].

Overexpression of the *c-MET* gene has been associated with unfavorable responses to treatment, for example in the therapy of *EGFR*-positive patients with tumors resistant to TKI [77].

### 5.5. Gliomas

Gliomas represent the most frequent type of *de novo* brain tumor, with a frequency reaching 30% of all brain tumors, including 80% of all nervous system [78]. The four-degree tumor aggressiveness WHO classification was proposed for Classification of Tumors of the Central Nervous System in the 2007. The newest, 5th version of this classification, considers not only histological analysis, but also genetic aberrations. This approach has contributed to the classification of cases of astrocytic and oligodendroglial tumors into one category, as mutations in the *IDH1/2* (isocitrate dehydrogenase (NADP (+)) 1/2) gene occur in both types. Further differentiation is based on unique genetic changes. Oligodendroglioma and anaplastic oligodendroglioma present the 1p/19q-codeletion, while astrocytoma presents mutations in the *ATRX* (ATRX chromatin remodeler) and *TP53* (tumor protein p53) genes [79,80].

Codeletion of 1p/19q, explained as the presence of a chromosomal deletion of the short arm of chromosome 1 and the long arm of chromosome 19 in the same cell, was considered for the first time as an oncological marker in neuronal tumors in 1994 [81]. Coexistence of 1p and 19q deletions is a result of an unbalanced translocation t(1;19) (q10;q10). The aberration can be detected using routine cytogenetic methods based on tumor cell cultures and G-banding (Giemsa-banding), but more frequently FISH on FFPE is used [13,15]. The nature of the translocation can be tested using the chromosome 1 alpha-satellite (CEP1) and *locus* 19q12 probes [22]. In the routine cytogenetic–molecular diagnostics, a kit of two probes is used: 1p36/1q25 and 19p13/19q13. Each set requires analysis of at least 100 non-overlapping nuclei for each pair of chromosomes. Interpretation of results is based on standards established for neuroblastoma by the International Society of Pediatric Oncology (SIOP). The calculated percentage of cells harboring the deletion has to be higher than 50% and ratio lower than 0.75%–0.8% for 1p36/1q25 as well as for 19q13/19p13 [79,82]. Alternative methods of detection of the codeletion include CGH (comparative genome hybridization) or loss of heterozygosity (LOH) analysis based on microsatellite sequences [79]. In addition to 1p19q aberrations, extra unbalances might occur, for example, trisomies of chromosomes 11, 17, 21, 22, and monosomies of chromosomes 14, 15, 18 [78].

Codeletion of 1p/19q plays a decisive role in distinguishing between oligodendroglioma and astrocytoma, which is especially helpful in histologically difficult cases. The genetic marker has also a prognostic value [15,79].

### 5.6. Breast Cancer

Breast cancer (BC) is the most frequent type of tumor identified in women [83]. Moreover, it is one of the main mortality factors in the world in this group of patients, with a five-year survival rate ranging from 57.9%–82% in Europe, depending on the country [84], with 77.2% in Poland [85].

The *ERBB2* (erb-b2 receptor tyrosine kinase) gene, frequently called *HER2*, is a proto-oncogene coding for a cell receptor involved in a few signal pathways responsible, e.g., for cell division and viability [83]. A significant abnormality of *HER2* is the occurrence of extra copies of this gene defined as amplification. A consequence of this aberration is over-expression of the protein which is found in approximately 22%–28% of BCs [86,87,88,89] and 8.2%–53.4% of gastric cancers [20,90,91]. While routine analysis of the protein may be conducted using IHC, the number of gene copies is assessed using the gold standard FISH on FFPE samples or CISH [11,19,37]. Status of the *HER2* gene is evaluated following the guidelines issued by the American Society of Clinical Oncology and the College of American Pathologists (ASCO-CAP), for the first time defined in 2007 [92]. According to the newest version of 2018, amplification is considered for *HER2*/CEP17 (centromere 17) ratios of ≥2.0, with at least 4 copies of the *HER2* gene per cell. For ratios of <2 and at least 6 copies of the *HER2* gene per cell, the final result is positive only for IHC3+ or when an additional analysis performed by a new observer on at least 20 cells confirms the same result (ratio of <2 and ≥6 *HER2* signals/nucleus) for IHC2+. A similar procedure relates to equivocal results as well, still defined as a combination of a ratio of <2 and *HER2*/CEP17 ≥4 and <6. In this situation, IHC3+ results again to facilitate the final diagnosis as *HER2*-positive. However, for IHC2+ results, only a result of an extra analysis of at least 20 cells with a ratio of ≥2.0 and HER2/CEP17>6 is consistent with *HER2*-amplified status of the gene [93]. This borderline situation occurs in about 10%–17% of FISH analyses [19].

Although the use of two differently labeled probes for the *HER2* l*ocus* and centromere 17 at the same time seems to be an optimal solution, results may not reflect only the amplification of *HER2* in the analyzed population of tumor cells, but also co-amplification of a bigger part of chromosome 17. The polysomy event, defined as at least 3 copies of the centromere of chromosome 17 per cell, is observed in 12%–46% of breast cancer cases [30,94,95]. Moreover, it is frequent in IHC2+ cases [20]. They are available other probes for *HER2* gene with a bigger distance between tested and control region, for instance, *RARA* (retinoic acid receptor alpha)—17q21.2, *RAI1* (retinoid acid-induced 1)—17p11.2, *TP53*–17p13.1 [19].

FISH testing of the *HER2* gene *locus* has a well-documented value for breast cancer patients. Personalized therapy can be used after assessing the *HER2* gene using one of the FDA-approved methods IHC, CISH or FISH, compared. Treatments include inhibitors or humanized monoclonal antibodies (e.g., pertuzumab, trastuzumab). Results stating overexpression or amplification of *HER2* are indications to start targeted treatment. In this case, personalized medicine contributes to the success of treatment, improves overall survival and extends the time to progression and metastasis. Moreover, thanks to focusing the therapy on *HER2*-positive patients, the general costs of breast cancer therapy are reduced [2,3,9,11,37,93,96,97]. 

### 5.7. Ovarian Cancer

Ovarian cancer is a heterogeneous type of tumor with multiple entities, the most typical of which are epithelial, germ cell and specialized stromal cell tumors. The first type, which reflects most of the cases, is divided into low-grade endometrioid cancer type I with gradual growth and beneficial prognosis and high-grade non-endometroid cancer type II with fast progression and adverse response to treatment. The leading form of the epithelial type is a very aggressive high-grade serous ovarian carcinoma (HGSOC) [24,98]. Its etiopathogenesis lies in mutations of the *TP53* gene, believed to be a primary event in carcinogenesis, but also in mutations in genes such as *BRCA1* (BRCA1 DNA repair associated) and *BRCA2* (BRCA2 DNA repair associated), involved in the reliable mechanism of double-stranded breaks (DSBs) repair called homologous recombination (HR), and finally in copy number alterations, including extra copies of the *CCNE1* (cyclin E1) gene [98]. The protein product of this gene, cyclin E, takes part in switching cell cycle between the G1 and S phases by activation of cyclin-dependent kinase 2 (Cdk2), phosphorylation of the Rb protein and E2f-1-dependent transcription [24]. Increased expression of *CCNE1* stimulates uncontrolled replication of DNA, amplification of centrosome and enhances chromosomal instability [98,99].

Assessment of the copy number of the *CCNE1* gene is based on FISH on FFPE using a *locus*-specific probe for a target gene located in 19q12 and a control region which is the centromere of the same chromosome (CEP19). At least 50 fully exposed nuclei undergo the analysis. A sample is considered as positive if the status of amplification understood as the *CCNE1*/CEP19 ratio of ≥2 occurs in more than 20% of tumor cells, or if ≥4 *CCNE1* copies are presented in ≥40% of analyzed cells, which is regarded as high polysomy [23]. What is interesting, the profile of the amplified gene *locus* varies between cases of clear cell carcinoma and high-grade serous carcinoma. In the former, extra copies are easy to count which suggests amplification of a double minute (dm) type, while in the latter, signals do not have sharp contours, and frequently are impossible to precise number assessing like in homogeneously staining region (hsr) type. These differences suggest the occurrence of two ways in which extra copies of the *CCNE1* gene are gained [23].

*CCNE1* testing is valuable for several reasons. Firstly, as overexpression occurs in clear-cell carcinoma cases, the test may be helpful in differentiation from other ovarian tumors based on endometriosis. Secondly, gains of *CCNE1* may be a predictor of short survival rate in clear-cell carcinoma. Thirdly, patients with identified gains of *CCNE1* may receive target-based therapy [23]. That last reason seems to play an important role due to the availability of therapeutic options. The common use of cytotoxic agents based on platinum is responsible for insensitivity to treatment if *CCNE1* amplification is present [24]. Searching for new molecules inhibiting CDKs, such as CDK2, may bring some benefits for patients with ovarian cancer [98].

### 5.8. Soft Tissues Sarcomas

Soft tissue sarcomas (STS) are uncommon cancers, with a frequency of 3.58–6.1/100,000 [100,101]. Their complex biology impedes the correct identification of tumor types in many cases. Therefore, both immunohistochemical and histological analyses are required. Although genetic testing is not the main diagnostic tool, it significantly supports the final diagnosis. The value of these tests has been approved in the WHO classification for many sarcomas in which crucial gene sequences have been identified, e.g., Ewing sarcoma (ES) [14].

At the molecular level, ES is characterized by rearrangements of the *EWSR1* (EWS RNA binding protein 1) gene [102]. The gene has several translocational partners which belong to the *ETS* family of transcriptional factors, such as the *FLI1* (Fli-1 proto-oncogene, ETS transcription factor) gene located in *locus* 11q24 [103], or the *ERG* (ETS transcription factor ERG) gene in *locus* 21q22. Translocations of the *EWSR1* gene with its partners are responsible for carcinogenesis: changes in cellular metabolism and proliferation, as well as apoptosis blockade [32,104,105].

Another sarcoma—synovial type (SS)—is represented by abnormalities of the *SS18* (SS18 subunit of BAF chromatin remodeling complex) gene, for example as a result of a t(X;18) (p11.2;q11.2) translocation. Gene partners such as *SSX1* (SSX family member 1) and *SSX2* (SSX family member 2) determine the histological subtype of SS: monophasic and biphasic, respectively. It has been observed that patients with the fusion *SYT–SSX1* have a longer survival [105,106,107,108,109].

In both mentioned sarcomas, assessing the gene status is possible using a break-apart probe and the FISH technique. Thanks to the structure of the probe, the demanded picture should present separation of fusion signals in more than 14 out of 50 analyzed tumor cells for the *EWRS1* gene and at least 10 cells for the *SS18* gene [14].

FISH testing based on recognition of gene rearrangements or specific gene fusions allows distinguishing pathological diagnosis, which is especially helpful in poorly differentiated sarcomas, e.g., some SS tumors may resemble small round cells of EWS, but genetically have a rearrangement of *SS18* gene. The correctness of diagnosis has a direct impact on the course of therapy and the patient’s prognosis [12,14,105].

Dermatofibrosarcoma protuberans (DFSP) is an uncommon tumor of mesenchymal tissues. In the newest WHO Classification of Soft Tissue Tumors issued in 2013, DFSP was assigned into the intermediate group of fibroblastic/myofibroblastic tumors. Recurrence of lesions is frequent and results in repeated surgeries. Metastases appear mostly in cases with a fibrosarcomatous component [110]. Diagnostics of this type of neoplasm is based on physical examination followed by morphological and immunohistochemical testing. However, since the discovery that a genetic abnormality underlies a great part of cases, genetic testing has become a helpful diagnostic procedure [26]. In 68%–96% of dermatofibrosarcoma protuberans cases, a translocation between chromosomes 17 and 22 occurs [26,33,111,112,113]. A result of this aberration is a fusion of the *COL1A1* (collagen type I alpha 1 chain) gene with the *PDGFB* (platelet-derived growth factor subunit B) gene [114]. The fusion results primarily in an enhanced expression of *PDGFB*, but also in a strong activation of the receptor of this ligand PDGFRB and stimulation of proliferation of mesenchymal cells [115]. The abnormality can be detected with the FISH method using a dual-color dual-fusion probe. When overlapping of signals of both differently labeled loci is observed as a yellow signal, the result is considered positive on condition that it affects at least 10% of analyzed cells. Alternatively, the *PDGFB locus* might be tested with the use of split type probe. Separation of signals complementary to the tested *locus* in at least 10% of cells confirms the rearrangement of that *locus* [26,33].

Diagnosis of the fusion *COL1A1–PDGFB* plays a crucial role in future therapeutic decisions as it is an indication for starting treatment based on imatinib mesylate. The inhibitor can be used in inoperable, metastatic and recurrent cases of dermatofibrosarcoma and seems to be an option as first-line treatment as well [116].

## 6. Conclusions

Established in the 1980s, fluorescence in situ hybridization is a technique useful in detecting various abnormalities, such as fusion of genes and genomic gains and deletions. Owing to gradually implemented improvements, the method has been yielding crucial information to be used not only in more precise patient diagnostics, but also in matching best therapies [39]. Some excellent examples include genetic tests of the *ALK* and *ROS1* genes performed in lung cancer required for TKI treatment. The ASCO recommendations of 2018 accept an IHC test of *ALK* in this neoplasia provided that the result is obviously positive or negative. For *ROS1*-positive and *ALK*-weak staining, the FISH method is proposed as decisive [117]. Other non-*in situ* methods, e.g., RT-PCR (reverse transcriptase-polymerase chain reaction) or NGS (next-generation sequencing) are also used, for instance for searching aberrations in lung cancer. The former method has a high sensitivity and specificity, but faces many issues related to isolation of ribonucleic acid derived from an FFPE sample or numerous gene partners. The latter seems superior as it requires a low amount of RNA, even from FFPE samples, and keeps sensitivity and specificity at the level of 100% [118]. Compared with the above mentioned IHC and FISH, high costs of chemistry and equipment and complex analysis and interpretation of results performed by a specialist in medical laboratory genetics still make NGS a possible supportive rather than routine technique [119]. To date, timeless and costless fluorescence in situ hybridization remains a gold standard, for example in NSCLC [31].

## Figures and Tables

**Table 1:** Comparison of immunohistochemistry (IHC), chromogenic in situ hybridization (CISH) and fluorescence in situ hybridization (FISH) techniques.

	IHC	CISH	FISH
**Concept of the Method**			
	assessment of protein expression using antigen-specific antibodies	assessment of chromogenic effect in an enzymatic reaction	assessment of chromosomal aberration using a fluorescent probe
**Advantages**			
**preparation**	- easy manual preparation [2] - automated preparation possible (e.g., Ventana BenchMark–USA) [7]	- easy manual preparation [8] - automated preparation possible (Ventana BenchMark—USA) [2,7] - permanent result of staining [2,5]	- easy manual preparation [8] - short preparation thanks to fast hybridization buffer - automated preparation possible (e.g., Ventana Medical System, Tucson, USA) [3]
**analysis**	- low-priced equipment for analysis (light microscope) [2] - automated analysis possible, e.g., ACIS, ChromaVision Medical Systems (San Juan Capistrano, CA) [3], or Applied Spectral Imaging, (Israel) - simple system of result evaluation (0, 1+, 2+, 3+) [9,10]	- low-priced equipment for analysis (light microscope) [2,5] - automated analysis possible (e.g., Applied Spectral Imaging, Israel) - assessment of copy-number alterations and cells morphology possible at the same time [2,5,11] - quantitative interpretation of result [2,11] - use of internal control possible [11]	- possible automatized scoring (e.g., Applied Spectral Imaging, Israel) - quantitative interpretation of result [2,8,10] - established cut-off values for probes [7,9,12,13,14,15]
**other**	- low-priced [2,16]	- low-priced [3]	- allows distinguishing *HER2* amplification from polysomy 17 (pseudoamplification) [9] - allows detecting rearrangement and deletion at the same time (*ALK*, *ROS1*) [7] - allows detecting rearrangement with many possible gene partners (*ALK*, *EWSR1*) [16] - presence of control cells (nonneoplastic) on the same slide (internal control of preparation) [3] - analysis of concordance of results between independent observers [2]
**Disadvantages**			
**preparation**	- differences in sensitivity and specificity caused by antibodies used, fixation methods [2,5,7]	- technical errors including fixation and digestion influence the final result [3]	- time-consuming when performed with standard chemicals [3] - gradual fluorescence weakening with time [5]
**analysis**	- semi-quantitative interpretation of results (subjective assessment) [3,17] - possible discrepancies between results (e.g., for score 2+) due to qualitative interpretation of result based on subjective judgment - necessity of re-evaluation of result of *HER2* (2+) using FISH [2] - false positive result of overexpression for 3+ due to polysomy 17 (in breast cancer) - necessity of re-evaluation of positive result of *ALK* using FISH (sometimes negative result as well) [18]	- no ratio result for amplification [3] - necessity of extra staining to exclude polysomy, e.g., of chromosome 17 [3] - possible problems with interpretation of fusion signals [7]	- specialized equipment (fluorescence microscope with a set of filters) - limited assessment of cell features (size and shape) [2] - possible discrepancies between independent observers in low-level amplification cases, equivocal case (*HER2*) [3] - possible discrepancies between independent observers in borderline distance between probe parts [10]
**other**	-	-	- higher costs compared with the other two methods [5,7,17]
	**Examples of solid tumors with use of the method**		
	- breast cancer [3,19] - gastric cancer [20] - lung cancer [16] - glioma [21,22] - ovarian cancer [23,24] - soft tissue sarcomas: EWS, SS, DFSP) [25,26]	- breast cancer [2,3,7] - gastric cancer [7] - lung cancer [7,27] - glioma [28] - soft tissue sarcomas: EWS, SS [29]	- breast cancer [3,9,11] - gastric cancer [30] - lung cancer [7,31] - glioma [22] - ovarian cancer [23,24] - soft tissue sarcomas: EWS, SS, DFSP [14,26,32,33]

**Table 2:** Summary of FISH technique value in solid tumors.

Type of Tumor	Diagnostic Value	Prognostic Value	Predictive Value	Available FISH Probe
Lung cancer ALK, ROS1	no	yes	yes crizotinib	▪ Vysis ALK Break Apart FISH Probe Kit (Abbott Molecular) [7,40] ▪ Vysis 6q22 *ROS1* Break Apart FISH Probe (Abbott Molecular) [41] ▪ ROS1 Break Apart FISH Probe (Empire Genomics)
Glioma co-deletion 1p19q	yes	yes	No	▪ Vysis LSI 1p36 SpectrumOrange/1q25 SpectrumGreen Probes and Vysis LSI 19q13 SpectrumOrange/19p13 SpectrumGreen Probes (Abbott Molecular) [13]
Breast cancer *HER2*	no	yes	yes trastuzumab, lapatinib	▪ PathVysion HER-2 DNA Probe Kit (Abbott Molecular) [37]
Ovarian cancer *CCNE1*	no	yes	yes platinum-based agents	▪ CCNE1/CEN19p FISH Probe (Abnova) [23]
Ewing sarcoma *EWSR1*	yes	yes	No	▪ Vysis EWSR1 Break Apart FISH Probe Kit (Abbott Molecular) [14]
Synovial sarcoma *SS18*	yes	yes	No	▪ Vysis SS18 Break Apart FISH Probe Kit (Abbot Molecular) [14]
Dermatofibrosarcoma protuberans *COL1A1-PDGFB*	no	yes	yes TKI (imatinib)	▪ SPEC COL1A1-PDGFB Dual Color Dual Fusion (ZytoLight) [42]

## Abbreviations

The following abbreviations are used in this manuscript:

ACMG	the American College of Medical Genetics and Genomics
ASCO-CAP	the American Society of Clinical Oncology and the College of American Pathologists
BC	breast cancer
Cdk2	cyclin-dependent kinase 2
CGH	comparative genome hybridization
CISH	chromogenic in situ hybridization
CTC	circulating tumor cells
DAB	3,3′-diaminobenzidine;
DFSP	dermatofibrosarcoma protuberans
DSBs	double-stranded breaks
*EGFR*	epidermal growth factor receptor
ES	Ewing sarcoma
*EZR*	Ezrin
FDA	U.S. Food and Drugs Administration
FFPE	formalin-fixed paraffin-embedded
FISH	fluorescence in situ hybridization
G-banding	Giemsa-banding
H&E	Hematoxylin and Eosin
HCl	hydrochloric acid
HGF	hepatocyte growth factor
HGSOC	high-grade serous ovarian carcinoma
HRP	horseradish peroxidase
HR	homologous recombination
hsr	homogeneously staining region
IHC	Immunohistochemistry
*KLC1*	kinesin light chain 1
LOH	loss of heterozygosity
MAPK	mitogen-activated protein kinases
MLPA	multiplex ligation probe amplification
mRNA	messenger ribonucleic acid
NCNN	the National Comprehensive Cancer Network;
NGS	next-generation sequencing
NSCLC	non-small cell lung cancer
*PDGFB*	platelet-derived growth factor beta
RT-PCR	reverse transcriptase-polymerase chain reaction
*SDC4*	syndecan 4
SIOP	the International Society of Pediatric Oncology
SS	synovial sarcoma
SSC	saline-sodium citrate
STS	soft tissue sarcomas
TKI	tyrosine kinase inhibitors
*TP53*	tumor protein 53
*TPM3*	tropomyosin 3
